# Anganwadi worker time use in Madhya Pradesh, India: a cross-sectional study

**DOI:** 10.1186/s12913-020-05857-4

**Published:** 2020-12-07

**Authors:** Anoop Jain, Dilys M. Walker, Rasmi Avula, Nadia Diamond-Smith, Lakshmi Gopalakrishnan, Purnima Menon, Sneha Nimmagadda, Sumeet R. Patil, Lia C. H. Fernald

**Affiliations:** 1 Harvard University, Boston, USA; 2 University of California San Francisco, San Francisco, USA; 3 International Food Policy Research Institute, Washington, DC, USA; 4 University of California Berkeley, Berkeley, USA; 5 NEERMAN, Center for Causal Research and Impact Evaluation, Mumbai, India

**Keywords:** Time use, Anganwadi workers, Maternal & Child Health, India, Madhya Pradesh

## Abstract

**Background:**

Anganwadi Workers (AWWs) are a group of 1.4 million community health workers that operate throughout rural India as a part of the Integrated Child Development Services program. AWWs are responsible for disseminating key health information regarding nutrition, family planning, and immunizations to the women and children in their catchment area, while maintaining detailed registers that track key beneficiary data, updates on health status, and supply inventory beneficiaries. There is a need to understand how AWWs spend their time on all of these activities given all of their responsibilities, and the factors that are associated with their time use.

**Methods:**

This cross-sectional study conducted in Madhya Pradesh, collected time use data from AWWs using a standard approach in which we asked participants how much time they spent on various activities. Additionally, we estimated a logistic regression model to elucidate what AWW characteristics are associated with time use.

**Results:**

We found that AWWs spend substantial amounts of time on administrative tasks, such as filling out their paper registers. Additionally, we explored the associations between various AWW characteristics and their likelihood of spending the expected amount of time on preschool work, filling out their registers, feeding children, and conducting home visits. We found a positive significant association between AWW education and their likelihood of filling out their registers.

**Conclusions:**

AWWs spend substantial amounts of time on administrative tasks, which could take away from their ability to spend time on providing direct care. Additionally, future research should explore why AWW characteristics matter and how such factors can be addressed to improve AWWs’ performance and should explore the associations between Anganwadi Center characteristics and AWW time use.

## Introduction

### Background

The role of community health workers (CHWs) has expanded considerably over the past 50 years, especially in low-and-middle-income countries (LMICs) [1]. A World Health Organization (WHO) report from 2016 highlights the chronic shortage of experienced health care workers in LMICs [2]. Many of these countries, for example, are simply unable to train and sustain an educated health workforce needed to ensure well-being and good health [2]. Thus, the burden of reducing morbidity and mortality, particularly in poor countries, often falls on the shoulders of a limited number of trained health care workers [2].

Task-shifting, a strategy proposed in 2004 for improving health outcomes, delegates the simplest health care tasks from health professionals (formally educated doctors, nurses, midwives, dentists, and psychologists) to local community members [3]. This approach could reduce the workload on professional health care workers while ensuring that community members have their basic health care needs met. Historically, CHWs perform a far more integral role than simply providing the most basic health services. For example, Kahssay et al. suggest that “the most important developmental or promotional role of the CHW is to act as a bridge between the community and the formal health services in all aspects of health development” [4]. In bridging this gap, CHWs can help ensure that their beneficiaries are receiving the care that they need.

Anganwadi Workers (AWWs) are a group of 1.4 million CHWs that operate throughout rural India as a part of the Integrated Child Development Services (ICDS) program, which has been running since 1975 under the Ministry of Women and Child Development (MWCD). AWWs are based at Anganwadi centers (AWCs), where they provide a variety of services to approximately 800–1000 children under the age of six, and pregnant and lactating mothers [5, 6]. AWWs are responsible for disseminating key health information regarding nutrition, family planning, and immunizations to the women and children in their catchment area, while maintaining detailed registers that track key beneficiary data, updates on health status, and supply inventory beneficiaries [7].

A number of studies have shown the positive impacts made by the ICDS AWW program. For example, one study used data from the 2005–2006 National Family Health Survey and found that ICDS improves linear growth for children from the poorest households [8]. Another study used data from the Demographic and Health Surveys (DHS) program from the same year and found that 0–2-year-old girls who receive intense supplementary feeding from ICDS are significantly taller than girls not receiving any supplementary feeding [9]. Reductions in the prevalence of underweight children have also been reported as a result of ICDS [10, 11]. However, service delivery gaps have also been reported. For example, several studies reported that many AWWs do not provide food to children or nutritional counseling to mothers when they are supposed to [12–14], that home visits are infrequent [15], and that the poorest households are often excluded from services [16].

AWWs are not unique in the problems of insufficient time being spent on key activities such as meal provision, health counselling, and home visits. For example, CHWs in a study in Ghana reported spending just only 10% of their daily work time on direct provision of services [17]. Similarly, Mazni et al. found that CHWs in their study in Tanzania spent only 57% of their daily time providing services [18]. Another study from Tanzania found that while CHWs spent 85% of their time between 9 am and 11 am providing health services, 90% of their time after 2 pm was spent on personal work [19].

A variety of factors could affect the amount of time CHWs, such as AWWs, spend on key activities and their overall performance. For example, a qualitative study conducted with CHWs in Nigeria, Burkina Faso, and Uganda found that while CHWs understood the voluntary nature of their work, they also felt that monetary incentives were critical to providing quality services [20]. CHWs from another qualitative study in India also reported that their pay was not commensurate with their effort and cited this as a cause of their lack of motivation to perform the duties of their job [21]. A number of other studies report similar findings that the lack of adequate or reliable remuneration leads to job dissatisfaction and stress, which undermines CHW performance [22–28].

Supervision, defined as a process through which supervisors monitor the work of peripheral workers and provide them with overall management [29], has also been found to alleviate some of the inefficiencies that stifle health worker productivity [24, 30–32]. Supervisor support, in the form of constructive feedback and goal setting, is also key to bolstering health worker productivity [33]. For example, results from a study in Ghana show that the effects of supervision on CHW productivity depend on how supportive the supervisors were [17]. Another study from Mali found that dedicated monthly supervision with feedback tailored to each CHW was associated with gains in CHW productivity [34].

The purpose of this paper was to build on this prior research using the context of AWWs in Madhya Pradesh, India. Our aims were to 1) describe how much time AWWs report spending on 11 major activities in their day-to-day work,^1^ 2) how this reported time deviates from the expected amount of time on the four activities deemed most important by the government (section 2.4.1), and 3) to elucidate what AWW characteristics are associated with AWWs spending the expected amount of time on these four activities. We hypothesized that there would be deviations from expected time spent on activities due to various AWW characteristics such as age and caste, along with the administrative burden placed on AWWs that might affect their time management.

## Methods

### Study area

This time use study is a part of a larger program evaluation of a mobile health application intervention called intended to improve AWW performance by automating the 11 registers they are required to complete,^2^ providing helpful reminders, and ensuring that they have access to essential resources such as automatic plotting of growth monitoring charts) and educational videos on birth preparedness, complementary feeding, family planning and sanitation. This study took place in Madhya Pradesh, where health care delivery is particularly difficult given the state’s geography (over 30% of its land area is covered by forest) [35]. Additionally, much like other states in India, the private sector dominates in Madhya Pradesh [36]. Furthermore, Madhya Pradesh has a high burden of under-five mortality at 69 per 1000 live births in 2015, where over 43% of children between the ages of 0–5 are stunted, 55% of pregnant women aged 15–49 are anemic, only 50% of children between the ages of 12–23 months are fully immunized, and only 8.3% of mothers have had full antenatal care [37].

### Participants and sampling

Our sample included 554 AWWs from six districts in Madhya Pradesh. These AWWs were a part of the evaluation endline survey, which took place during December 2018 and January 2019. A full description of the evaluation sampling strategy can be found in previously published work [38]. A full list of AWW characteristics can be found in Table 1.

**Table 1:** AWW Characteristics

	Category	Number	Percentage
**Caste**	Scheduled Caste	52	9.40%
	Scheduled Tribe	267	48.20%
	Other Backwards Class	126	22.70%
	General Caste	109	19.70%
	Total	554	100.00%
**Experience**	< 10 years	174	31.40%
	10–18 years	242	43.70%
	> 19 years	138	24.90%
	Total	554	100.00%
**Education**	Below 11th Grade	261	47.10%
	Above 11th	293	52.90%
	Total	554	100.00%
**Age**	< 36 years	204	36.80%
	36–44 years	169	30.50%
	> 44 years	181	32.70%
	Total	554	100.00%
**Other Work**	Yes	29	5.20%
	No	525	94.80%
	Total	554	100.00%
**Family Size**	< 4 members	69	12.50%
	4–7 members	310	55.90%
	> 7 members	175	31.60%
	Total	554	100.00%
**Has a helper**	Yes	463	83.60%
	No	91	16.40%
**Wealth**^**a**^	**Median**	**Min**	**Max**
	17	5	29

^a^The variable ‘Wealth’ is an additive measure of 31 unique binary variables, each of which represents a different household asset such as animals and livestock and durable goods

### Measurement tool

We collected time use data from AWWs using a standard approach, which has been used in several previous studies. For example, this method has previously been used in the Women’s Empowerment in Agriculture survey conducted in Uganda and Bangladesh [39]. Similarly, Kahneman et al. used a version of stylized questions in their study that examined how 1108 women in the United States spent their time [40]. This is a method that involves asking respondents questions about how much time they spend conducting various activities as opposed to asking respondents how they spent their time in a given time range [41]. Trained interviewers questioned each AWW at the end of the day they visited the center about the time spent conducting each pre-specified activity on the day of the interview. For example, AWWs were asked questions such as, “how much time did you spend updating paper registers?” and “how much time did you spend conducting growth monitoring?”. Responses were recorded in terms of the number of hours and minutes spent on the activity. Thus, we did not ask respondents to recall what they did in pre-specified time intervals.

Developing the final list of activities was an iterative process, which began in 2018 with a pilot study. We first shadowed 36 AWWs and noted all of their daily activities, and the amount of time spent doing each one. This pilot study helped us develop a list of 64 key activities that could be grouped in to nine categories. We also used the guidelines issued by the Ministry of Women and Child Development to gain an understanding about the key activities that AWWs are responsible for executing on a daily basis, along with the amount of time that should be spent on these activities [42].

Informed consent was obtained from all participants before any interviews were conducted. Study protocols have been reviewed and approved by institutional review boards at the University of California, Berkeley (reference number: 2016-08-9092), and the India-based Suraksha Independent Ethics Committee (protocol number: 2016-08-9092). The trial is registered with the ISRCTN registry (10.1186/ISRCTN83902145).

### Measures

#### Outcomes

We studied eleven outcomes which are the amount of time spent (in minutes) on eleven activities listed in Table 2 based on the self-reports by the AWWs. The Ministry of Women and Child Development (MWCD) have identified the first four activities as the core tasks and have specified the expected amount of time that should be spent on each one. For example, AWWs are expected to spend 60 min conducting home visits, 30 min feeding children at the Anganwadi Centre, 120 min on preschool work, and 30 min on paper registers every day. For each of these four activities, therefore, we created a binary outcome – 1 if an AWW spent the expected time or more on the activity, and 0 otherwise.

**Table 2:** Summary of outcome variables (in minutes), *n* = 554

Time Spent On	Median	Max	Mean	Percent of Total Mean Time
**Home Visits**	30	120	32.4	9%
**Feeding**	60	120	52.8	15%
**Preschool Work**^a^	90	240	95.3	26%
**Paper Register Work**	47.5	180	51.1	14%
**Updating Mobile App**	0	180	13.2	4%
**Childcare**	30	180	41.4	11%
**Growth Monitoring**	0	120	9.3	3%
**Meetings**	0	240	10.2	3%
**Other ICDS Work**	10	240	22	6%
**Non-ICDS Work**	0	270	13.2	4%
**Resting**	0	210	18.5	5%
**Total**			359.4	

^a^Preschool work refers to the time during the day when the AWW functions as a preschool teacher

#### Independent variables

We modeled AWW caste^3^ (tertiles, bottom as reference), age, education (above/below 11th grade), years of experience working as an AWW (tertiles, bottom as reference), whether or not they work another job (yes/no), whether or not they have a helper (yes/no), wealth (additive index of 30 components), and family size as defined as the number of all family members living together (tertiles, bottom as reference). These variables were selected based on our hypotheses about factors that might be associated with the primary outcomes.

### Analytical approach

The general form of the model that we estimated for understanding what factors (**X**) are associated with spending the expected amount of time on a core activity was: logit (π_ij_) = β_0_ + β_1_**X**_ij_ + ε_ij_, where π_ij_ represents the odds for a given outcome for AWW i in village j. We exponentiated our results so that estimates the odds of spending the expected time, or more, on the given activity for all of the independent variables specified above.

## Results

### Sample characteristics

Among the 260 AWWs with the mobile application and 294 AWWs without the application in our sample, the largest share belonged to the Scheduled Tribe category (44%) (Table 1). In our sample, 36% of AWWs had between 10 and 18 years of experience working as AWWs. There was an even split in educational attainment, and the average age of AWWs in our sample was 40. Furthermore, 94% of the AWWs reported having no other form of work.

### Overall time use

On average, the AWWs in our sample spent a total of 360 min (6 h) working per day, which is the number of hours AWWs are expected to work (Table 2). The activity that took up the most time during the day was preschool work (26%); AWWs spent the least amount of time attending meetings and on growth monitoring (3% of their total workday on each activity). Home visits accounted for 9% of daily work time, while feeding and paper register work took up 15 and 14% of daily work time, respectively. Other work related to ICDS (opening and closing the AWW, for example) accounted for 6% of daily work time, while 5% of daily work time was spent resting. Overall, the AWWs in our sample spent a total of 52% of their daily time directly serving children (26% on preschool work, 15% on feeding, and 11% on childcare).

### Home visits

In our sample, 331 AWWs reported conducting home visits on the day of our data collection. These AWWs reported spending an average of 32 min conducting home visits, which is 28 min less than the expected daily time specified by the government (Table 3). The majority (65%) of AWWs in our sample spent less than the expected amount of daily time conducting home visits. We found that higher odds of spending the expected amount of time or more on home visits was significantly associated with being Scheduled Tribe (odds-ratio: 1.69, 95% CI 0.97, 2.94) after controlling for all other covariates (Table 4).

**Table 3:** Percent of AWWs that spent expected daily time on home visits, feeding, preschool work, and paper registers

Activity	Expected daily time (mins)	Percent below	Percent above
**Home visits**	60	65.7%	34.3%
**Feeding**	30	10.3%	89.7%
**Preschool work**	120	54.9%	45.1%
**Paper registers**	30	17%	83%

**Table 4:** Regression results^a^

	Home Visits	Feeding	Preschool Work	Paper Register Work
**Scheduled caste**	1.84	4.08	0.81	0.65
	(0.87, 3.89)	(0.49, 34.2)	(0.39, 1.65)	(0.21, 2.01)
**Scheduled tribe**	1.69*	0.48*	0.76	0.34**
	(0.97, 2.94)	(0.20, 1.11)	(0.46, 1.28)	(0.14, 0.79)
**Other backward class**	1.17	1.48	0.81	0.96
	(0.64, 2.13)	(0.52, 4.25)	(0.47, 1.41)	(0.35, 2.58)
**Above 11th grade**	1.26	0.75	1.29	2.13***
	(0.84, 1.82)	(0.39, 1.44)	(0.87, 1.91)	(1.22, 3.73)
**36–44 years old**	1.12	0.78	1.29	1.59
	(0.69, 1.83)	(0.36, 1.68)	(0.81, 2.05)	(0.82, 3.08)
**Above 44 years old**	1.43	0.65	1.10	1.06
	(0.80, 2.53)	(0.26, 1.62)	(0.64, 1.92)	(0.52, 2.18)
**10–18 years of experience**	1.45	1.35	0.82	1.37
	(0.87, 2.42)	(0.61, 2.96)	(0.51, 1.33)	(0.69, 2.74)
**Above 19 years of experience**	1.13	1.58	0.81	0.95
	(0.59, 2.14)	(0.57, 4.38)	(0.44, 1.49)	(0.41, 2.16)
**Other work**	1.70	2.61	0.45	1.03
	(0.78, 3.73)	(0.34, 20.21)	(0.19, 1.05)	(0.32, 3.27)
**Wealth**	0.97	1.02	1.03	0.96
	(0.92, 1.02)	(0.94, 1.11)	(0.98, 1.09)	(0.89, 1.02)
**4–7 family members**	1.24	1.18	1.05	0.76
	(0.68, 2.25)	(0.48, 2.92)	(0.61, 1.82)	(0.34, 1.70)
**Above 7 family members**	1.45	1.20	1.23	0.89
	(0.76, 2.77)	(0.45, 3.21)	(0.67, 2.26)	(0.37, 2.14)
**Has helper**	0.78	1.09	1.13	1.84**
	(0.48, 1.28)	(0.53, 2.25)	(0.69, 1.81)	(1.03, 3.25)
**Constant**	0.34	7.19**	0.46	8.19**
	(0.09, 1.23)	(1.01, 51.48)	(0.14, 1.53)	(1.44, 46.69)
**Observations**	554	554	554	554

^a^Multivariate logistic regression results

(95% CI))

*** *p* < 0.01, ** *p* < 0.05, * *p* < 0.1

### Feeding

AWWs in our sample reported spending an average of 52 min on feeding, which is 22 min more than the expected daily time. The majority (90%) of AWWs in our sample spent more than the expected amount of daily time on feeding (Table 3). We did not find any significant associations for feeding.

### Preschool work

AWWs in our sample reported spending an average of 95 min per day on preschool work, which is 25 min less than the expected daily time. There was a fairly even split between the percent of AWWs who spent below and above the expected amount of daily time on preschool work (55 and 45% respectively) (Table 3). We did not find any significant associations for preschool work.

### Paper registers

AWWs in our sample reported spending an average of 51 min filling out their paper registers, which is 21 min more than the expected daily time. The majority (83%) of AWWs in our sample spent more than the expected amount of daily time on paper registers (Table 3). We also found AWW from a Scheduled Tribe had lower odds of spending the expected amount of time or more on paper registers (odds-ratio: 0.34, 95% CI 0.14, 0.79) (Table 4), after controlling for all other covariates. We found that higher odds of spending the expected amount of time or more on paper registers associated with the AWW having attained above the median level of education (11th grade) (odds-ratio: 2.14, 95% CI 1.22, 3.73) (Table 4), after controlling for all other covariates. We did not find any other significant associations for paper registers.

## Discussion

Overall, our findings suggest that caste, education, having helpers and having other jobs were the associated time use. Specifically, our findings show no significant associations between AWW age and the likelihood that they spend the expected amount of time on home visits, feeding, preschool work, or paper register work. Scheduled Caste AWWs were more likely to spend the expected amount of time conducting home visits compared to general caste AWWs but were less likely to spend the expected amount of time on feeding and paper register work than their general caste counterparts. AWWs with helpers were almost twice as likely to spend the expected amount of time on paper register work compared to AWWs without helpers. Furthermore, AWWs who had at least an 11th grade education were more than twice as likely to spend the expected amount of time on paper register work than AWWs with less than an 11th grade education.

Additionally, our results indicate that on average, the AWWs in our sample spent a total of 52% of their daily time directly serving children which is encouraging given that children are a core focus of the ICDS program. However, the AWWs in our sample spent only 9% of their daily work time conducting home visits and 3% of their time on growth monitoring. Conducting home visits, and measuring child height and weight, are essential as they help ensure that children are meeting their growth targets. There are several possible explanations for these findings. For example, AWCs often do not have growth monitoring charts available. In fact, 30 and 21% of AWCs in two separate studies did not have growth charts present, respectively [43, 44], while 49% of the AWCs in our sample did not have growth charts. Another reason is that AWWs do not measure every day, and thus enumerators may not have visited a given AWW the day she was supposed to conduct child measurements. Similarly, AWWs may not have been required to conduct any home visits on the day enumerators visited them; ICDS has also issued guidelines on when children, and pregnant and lactating mothers need to be visited at home and AWWs often group multiple visits on a single day as per this schedule.

Furthermore, 83% of the AWWs in our sample reported spending more than the expected amount of time on filling out paper registers. This could be an indication of the fact that the expected amount of time that AWWs should spend on activities such as paper registers could be unrealistic, especially given the fact that each AWW is typically responsible for serving approximately 1000 beneficiaries [43] and maintaining eleven registers. Spending so much time on completing registers is also evidence of how CHWs often feel compelled to spend time reporting and evaluating in order to help produce ‘numerical narratives’ to demonstrate successful public health programs as imagined by their superiors [44]. Such stringent reporting guidelines and requirements have been found to undermine an AWW’s ability to engage in activities that would help them develop a thorough understanding of the health needs of their beneficiaries (via activities such as home visits, for example) [45]. The reporting requirements in the context of this study were further compounded by the fact that AWWs equipped with the mobile application were also required to use paper registers as a failsafe.

Various AWW characteristics were associated with the amount of time that they spend on essential activities such as home visits, feeding children, register work, and preschool work. For example, AWW caste is a complex concept because it relates to social marginalization, educational qualifications, as well as socio-economic status. Stifled educational opportunities early in life could explain why low caste AWWs spend less time filling out paper registers than higher caste AWWs. Additionally, caste divisions within a community could undermine an AWWs ability to perform her job (if she is of low caste) [46]. Perhaps this could help explain why the Scheduled Tribe (historically marginalized group) AWWs in our study are less likely to spend the expected amount of time on feeding. Perhaps, they serve smaller population than general caste AWWs and thus spend less time doing paper register work when compared to general caste AWWs. On the other hand, Scheduled Tribe AWWs spent much more time on home visits perhaps because they serve tribal areas which can be spread out requiring more travel time. The focus of this study was not to identify how caste or other socio-economics characteristics play a role in AWW performance and we don’t have substantive data to explore such reasons further. However, this can be a useful future research area.

Additionally, AWWs in some parts of India have reported significant stress and job dissatisfaction [47], and often complain about being overworked, job insecurity, and the lack of proper AWC infrastructure [48, 49]. These factors, coupled with the fact that AWWs often report delays in being paid their salaries [50] could undermine an AWW’s motivation to spend adequate amounts of time performing her duties, and thus their impact. In fact, performance pay for AWWs has been found to significantly reduce underweight prevalence, thus demonstrating that AWWs perform better when they are paid appropriately [51]. The fact that AWW salaries are delayed might also be why some AWWs have to take up other jobs, which could explain why they are able to spend less time performing key tasks such as preschool work. This also raises the question about the extent to which the tasks performed by other groups of CHWs in India, such as Auxiliary Nurse-Midwifes and Accredited Social Health Workers and AWW helpers (95% of AWWs in our sample reported having a helper) might complement the work performed by AWWs. Our study only included AWWs, but ANMs, ASHAs, and helpers could be making up for work not done by AWWs in a given village, something that should be further studied.

There are several limitations to this study. First, as noted above, we collected data about how AWWs spent their time during one presumably representative day with no direct observation. This approach is prone to error given that it is asking respondents to recall how much time they spent on various activities, pointing to a flaw in self-reported data [52]. Furthermore, self-reported data could be problematic given top down reporting pressures, which have been found to create a drive for ‘right’ numbers leading to inaccurate reporting [53] Next, our outcomes were constructed as binary variables, indicating only whether an AWW spent above or below the expected amount of time on an activity. This approach did not capture whether or not the AWWs interviewed felt as though they had completed their necessary tasks of the day, the efficiency with which tasks were executed, or their motivation to complete a task, which could also influence time spent on activities. At a certain point, spending too much or too little time on an activity might be problematic. Spending too much time could take away from an AWW’s ability to spend time on other essential tasks, while not spending enough time on certain tasks could be associated with poor outcomes. Therefore, time use, while useful, is not necessarily a performance metric. Additionally, our survey did not capture data about AWW duties and responsibilities in her own home. AWWs with a greater burden of responsibilities in their own homes might be less able to spend the expected amount of time on their duties in the AWC. It is also unclear whether the government’s recommendations for expected time to be spent per activity are based in evidence. Our results show that perhaps future research is needed to better understand the appropriate amounts of times that should be spent on activities. Finally, we only collected data from each respondent for 1 day due to logistical constraints. However, it is more common to collect at least 2 days’ worth of data for reliability issues, and in order to capture data on less frequently occurring activities [41]. In the context of this study, this meant that we only collected time use data on home visits from 331 AWWs.

Despite these limitations, to our knowledge, this is perhaps the only study which has looked at how AWWs spend their time in a diverse sample of more than 400 villages from six districts and assessed their time use against government guidelines. We have also identified that certain AWW socio-demographic characteristics are strongly associated with their time-use outcomes which can guide future research, to study how and why they matter, or direct training and AWW support efforts.

## Conclusion

Overall, we find that AWWs spend a big share of their work time on filling out their paper registers even when a smartphone-based application is available, and they had been given the option to not use the paper registers. This could take away from their ability to spend the expected amount of time conducting home visits, and thus serving adolescent girls, pregnant women, and lactating mothers, and is something that can be addressed by the government. Second, we find that the current guidelines on expected time spent on core tasks may need to be revised to better account for the work realities of AWWs. For example, conducting home-visits every-day is not even needed as per ICDS’s own home-visit schedule and AWWs can combine multiple visits on a single day to save on travel time. Perhaps a newer set of guidelines that recognize varied work conditions faced by the AWWs and provide guidance and training resource on how to effectively plan and manage daily workload would be a useful resource, especially as the paper registers are phased out across the country. Additionally, ICDS should ensure that AWCs are equipped with all the necessary supplies and tools for AWWs to perform their tasks. For example, if AWWs had reliable access to records from the mobile application, they may not have needed to also maintain paper registers as a failsafe. Finally, immutable characteristics such as caste are significant predictors of an AWW’s ability to spend the expected amount of time on various activities. Future research can explore why these characteristics matter and how such factors can be addressed to improve AWWs’ performance.

## Data Availability

The datasets analyzed during the current study are not publicly available given that the research team has not completed its analysis but are available from the corresponding author on reasonable request.

## Abbreviations

AWWs: Anganwadi workers
CHWs: Community health workers
LMICs: Low-and-middle-income countries
WHO: World Health Organization
ICDS: Integrated Child Development Services
MWCD: Ministry of Women and Child Development
AWCs: Anganwadi Centers
DHS: Demographic and Health Surveys

## Footnotes

1 From here forward, work refers to Anganwadi work, not the household work that AWWs are also likely to be engaged in

2 At the time of this survey, none of the AWWs in our sample had given up their paper registers as they were still being used in a backup capacity

3 Scheduled tribe, scheduled caste, other backward class, and general caste are the four broad caste categories used in India’s system of affirmative action [41]. We used general caste as the reference category. Historically, scheduled castes and scheduled tribes have been the most marginalized groups in India.
